# A Study on Chinese Audience’s Receptive Behavior towards Chinese and Western Cultural Hybridity Films Based on Grounded Theory—Taking Disney’s Animated Film *Turning Red* as an Example

**DOI:** 10.3390/bs13020135

**Published:** 2023-02-06

**Authors:** Rui Chen, Yi Liu

**Affiliations:** 1 School of Literature and Journalism, Xihua University, Chengdu 610039, China; 2 Research Institute of International of Economics and Management, Xihua University, Chengdu 610039, China

**Keywords:** cultural hybridity, the third space, cross-cultural acceptance behavior, grounded theory, animated film

## Abstract

For a long time, Chinese audiences have not had a high opinion of hybrid Chinese and Western movies. However, the unanimous praise for *Turning Red* in China se ems to have reversed this situation. In order to verify whether the attitudinal behavior of Chinese audiences toward the film’s hybridization of Chinese and Western cultures has changed, this study collected textual materials reflecting the Chinese audience’s receptive attitudes toward the film: Douban reviews, short reviews, questionnaires and Mtime.com reviews. Through a grounded study of 664,312 words, a total of 16 initial categories and four main categories were obtained. Finally, a cognitive–emotional–attitudinal mechanism model was formed to explain the audience’s receptive behavior process. The study found that Chinese audiences’ positive reception of *Turning Red* comes more from the fact that the film touches on personal emotions and focuses on a series of issues such as growing up, family, and gender, with intergenerational conflict as the core. The audience achieves self-projection and empathy while watching the film, rather than recognizing the Chinese culture presented therein. On this basis, the research further found that the internal structure of the current cultural hybridity has not changed greatly. The reason audiences do not give a high evaluation of cultural hybridity films lies in the lack of conscious distinction between the hybridity culture and the local culture. At the same time, in terms of cross-cultural creation, we should abandon the blind pursuit of cultural symbols, take root in cultural soil and then pay attention to more specific problems. This study reveals that the key factor affecting the audience’s receptive behavior toward cultural hybridity films is not necessarily the performance of local culture, which is of great significance for establishing new evaluation criteria.

## 1. Introduction

In March 2022, the animated film *Turning Red*, produced by Disney’s Pixar unit, went online for streaming on Disney+, and even if it did not have a theatrical release, it still garnered much attention. Currently, it scores 8.2 on Douban, 7.0 on IMDb and 94% on Rotten Tomatoes, making it a hit on major review websites both at home and abroad. *Turning Red* is directed by Chinese Canadian Domee Shi, who previously won the Best Animated Short Film Award at the 91st Academy Awards for her animated short film *Bao*. *Turning Red*. She continues to maintain the focus of *Bao* on the parent–child relationship, but turns the visual focus from the mother to the child. It focuses on the repression and resistance of adolescent girls in their growth from a uniquely female perspective, reflecting the fetters of native families in East Asian society. Due to the diversity of the cultural backgrounds of the director and her team, the film brings together a large number of exchanges and collisions between Chinese and Western cultures, including the narrative style of classical Chinese mythology and Eastern cultural elements, as well as the universal values of love and reconciliation in the West. Hollywood films are integrated into the Chinese context, showing the complex interaction between the locals and the world from different cultural backgrounds [1].

In order to globalize and popularize their cultural products, and also to respond to the call for increased multiculturalism, Disney’s cultural fusion of countries and regions in recent years—creating films from a large number of different regions to create amorous feelings towards these films in these regions, such as *Coco*, *Black Panther*, *Raya and The Last Dragon,* and so on—aimed to release the potential of our imagination on a global scale [2]. As the world’s largest film consumption market, China has huge market potential, and at the same time, Disney has decided to focus on China due to its profound cultural resources. As early as 1998, Disney released the animated film *Mulan*, adapted from an ancient Chinese folk poem, *The Song of Mulan*. In 2020, Disney released a live-action version of *Mulan*, which injected the connotations of the new era into the story. In 2021 and 2022, Disney-owned Marvel and Pixar released *Shang-Chi and the Legend of the Ten Rings* and *Turning Red*, respectively, showing Disney’s exploration of more dimensions and broader thinking regarding Chinese culture. In addition, other films owned by Hollywood and other Western film companies, such as DreamWorks, Warner Bros and A24, have also been released, including the *Kung Fu Panda* series, *Crazy Rich Asians*, *The Farewell*, *Everything Everywhere All at Once,* and other works filled with the integration and encounters of Chinese and Western culture. These works combine local and foreign cultures, making the culture more colorful [3]. While globalization leads to the generalization of culture, cultural hybridity also occurs simultaneously [4]. This means that a cultural form is recombined with a new form in a new practice [5], which leads to the final form of telling a cultural story from one culture with the narrative mode of another culture.

Therefore, when Chinese stories filmed by Western media are introduced in China, there is a situation of “acclimation to the soil”. Generally speaking, compared with the non-collectivist social consumers of the United States, Chinese consumers, under collectivism, make fewer negative comments and have higher final evaluations on the same series of products [6]. However, when we compare the scores of these works on Chinese and Western mainstream film review websites (Douban and IMDb), as shown in Table 1, we find that the Douban rating of the same work is almost always lower than the IMDb score; that is, the acceptance of these works in China is lower than in Western countries. We can preliminarily judge that Chinese audiences resist films that hybridize Chinese and Western cultures. For example, *Crazy Rich Asians* was the highest-grossing romantic comedy of the decade in North America, while it was met with a lukewarm reception in China. Another example is the USD 200 million *Mulan*, which only grossed USD 40 million in China. Moreover, the first ever Marvel movie with a Chinese American superhero, *Shang-Chi and the Legend of the Ten Rings*, which grossed over USD 440 million, was not as beloved and popular with Chinese audiences as other Marvel heroes. Because of the cultural and political differences between the East and the West, the horizons of audiences’ expectations are different, and there is clear incommensurability between the East and the West in media products and consumption [7].

*Turning Red* is an exception, as it is the only work whose score on Douban is higher than that on IMDb. For a film made by a foreigner and containing Chinese culture, it has even gained higher reviews and deeper resonance in China. Could this be a sign that Chinese audiences have changed their receptive behavior towards the hybridization of Chinese and Western cultures? Further, when the audience is confronted with the familiar culture as “the other”, how will the audience perceive and accept this culture? Based on the unique example of *Turning Red*, we believe that this study is novel and original and that it will reveal insights into the complexity of the research subject. We also hope that this research will lead to substantive recommendations for the creation and dissemination of cultural hybridity, and its potential business and managerial implications.

## 2. Literature Review

### 2.1. Chinese Stories and Images of Chinese People in Western Films and Television Productions

The shaping of Chinese stories and images in Western films and television has roughly gone through three stages. The first stage is the representation of Fu Manchu and Charlie Chan, the typical characters of the first half of the last century. One of them is a representative of evil; the other is a symbol of obedience. These two characters are like “Satan and the family minister”, regulate the change of Chinese identity through a racist mechanism [8], and the image of China is acted out by this binary oppositional structure [9]. In the second half of the twentieth century, from the *Bruce Lee* series to *Crouching Tiger, Hidden Dragon*, from *The Wedding Banquet* to *the Joy Luck Club*, martial arts had been prominent, and the suffering of immigrants was told. At that time, the films were more about the display and reflection of a single culture and lacked the interweaving and communication of Chinese and Western cultures.

It was not until 1998, starting with the Disney animated film *Mulan*, that Western production companies began to create stories by appropriating Chinese culture. Chinese characters gradually became the main characters of the stories, from marginal supporting and flat characters, and their representation became three-dimensional. Moreover, these characters were thoroughly molded into the typical characters of the typical Western narrative mode. At the same time, the story’s content is less about a curious landscape and more about common problems. For example, *Crazy Rich Asians* imbues the romantic comedy template with a new meditation on the changing scenes of cross-cultural communication [10], while *The Farewell* explores different views of life and death in Chinese and Western cultures. In *Turning Red*, the focus is on the parent–child relationship and the growth of adolescent girls.

However, even with these changes, as China remains the cultural “other”, the stereotypes created by the “imagined other” in the work are still inevitable. Cross-cultural appropriation also transfers a society’s cultural anxiety to the gendered body of the other [11]. Moreover, inaccurate representations of minorities are both dangerous and problematic for out-group directors because they retain negative racial stereotypes rather than trying to resist and challenge them [12]. Instead of innovating, these works of art, directors frequently sell stereotypes and perpetuate traditional ideas [13]. In the works, these characters are either over- or under-constructed [14]. Although this sense of offense can be weakened by the advantages of films [15], some stereotypes are still retained (such as being weak, timid, and nerdy), which may affect the identity development of adolescents and the interaction between groups [16].

### 2.2. Cross-Cultural Audience Acceptance

Viewers’ perceptions of media content are influenced by a complex interactions of systems, environments, and human factors, in which human factors play a key role [17]. Intercultural awareness is required for intercultural communication and acceptance of film and television. Cross-cultural awareness is the basis for communication with other cultures, which emphasizes the awareness of different cultures’ values, beliefs, and perceptions [18]. Generally speaking, because cross-cultural communication is often related to the incompatibility between the host country and the traditional culture [19], audiences are in different social and cultural contexts, and different reading stances will lead to differences in acceptance [20]. As a result, cultural distance and cultural discount will be measured in cross-cultural communication of cultural products [21]. There is usually a positive correlation between cultural distance and cultural discount [22] related to cultural and structural differences such as language, history, style, and background [23].

The intervention of new media has brought about important changes in cross-cultural communication, and some classical cross-cultural theories need to be re-examined [24]. Cultural works with cultural hybridity, for example, differ from those envisioned by cultural distance. Despite similar or close cultural beliefs, a negative gaze within the same race is still possible [25]. In theory, their cultural content is closer to the Chinese audience, and the cultural distance is smaller, but they do not achieve a satisfactory reception effect.

If they have distinct ethnic characteristics, there will be group acceptance differences. Films dominated by a certain ethnic group will receive special attention from that group, especially those with a strong ethnic identity [26]. However, these Asian groups presenting their own stories do not believe that the current works show the obvious progress of Western media, nor can they reach a consensus among their dispersed groups [27]. Therefore, they strive to transcend and resist the restrictions of various communication platforms to adequately express their dissatisfaction with the current media environment [28].

The reception of these works by local Chinese audiences is more negative because, although these films focus on showing a large amount of Chinese culture and Chinese elements, it is still a Western narrative structure. Of course, it is not that Chinese audiences do not like the narrative logic of Western commercial blockbusters; otherwise, the Marvel hero series, Disney princess series, and so on would not be popular in China. It is just that when Chinese culture is involved in these works, Chinese audiences are more cautious, unconsciously comparing them with their real cultural environment. As a result, it is often found that there is a difference between the China shown in the film and the China they perceive. Cultural appropriation and stereotypes are full of them. The presentation of Chinese culture in films is limited to the superficial differences between folk customs and cultural landscapes, but it fails to touch real cultural connotations [29]. Therefore, these films do not have the effect of cultural export but just apply Oriental characters to Western values. Such works, which are empty of Oriental cultural symbols but lack the core of Oriental culture, have always prevented Chinese audiences from achieving a cultural identity. This also vaguely reveals the problems in the concrete expression of the current hybridization of Chinese and Western cultures.

### 2.3. Cultural Hybridity

The output of culture is not unidirectional; different cultures infiltrate each other in the process of contact, and cultural hybridity is put forward on this basis. The cultural hybridity proposed by Homi Bhabha, a scholar of post-colonialism, aims to abandon the previous research paradigm of binary opposition. Cultural hybridity refers to a state in which no culture can exist purely while not being influenced by other cultures [30]. Hybridity means the reconfiguration and reinterpretation of ideas, institutions, and practices when the West and the East meet, when the global meets the local [31]. The idea of cultural hybridity is that there is no hierarchy between primitive and heterogeneous cultures [32] and that the transculturality between different cultures leads to a “third space”, where boundaries are removed, and poles are moved, thereby creating hybrid identities [33].

Cultural hybridity, instead of cultural integration, is the key in that it holds that two kinds of cultures are in a two-way flow and influence each other. No one is attached to those who dominated in order to bring about a dynamic shift in the "third space." Cultural hybridity is driven by the market [34]. Some cultures have successfully achieved global popularity through cultural hybridity, such as the K-pop trend in South Korea [35]. However, the K-POP cultural hybridization process is more complicated; it positions new music content in Europe or other places by modifying the Korean content, and then re-release it globally, namely globalization–localization–re-globalization [36].

The theory of cultural hybridity is an exploration and a way of thinking developed by postcolonial scholars when seeking equality in terms of racial and gender identity. Many people have also questioned it since its birth. Although the essentialist view of culture easily leads to stereotypes, the extreme postcolonial theory of cultural hybridity completely rejects cultural differences [37]. Moreover, empirical studies have found that cultural hybridization has great potential to achieve adaptation, interaction, or collision in different contexts [38]. The West seriously influences the process of native cultural hybridization, and the newly created native cultural products mostly represent Western culture. Instead of unique local cultures, cultural hybridization cannot fully explain local culture due to the taming of power relations [39]. Therefore, the fusion and hybrid characteristics of the current cultural form and cultural hybridity may only exist in abstract ideas but lack a sufficient empirical basis [40]. When Western culture is mixed and hybridized with the cultures of other countries and regions, the final pattern is almost always that Western culture provides “universal” value, while non-Western culture provides exotic sensory enjoyment [41]. The previous attitude of Chinese audiences towards Chinese and Western cultural works also verifies this point. The cultural offense taken by Chinese audiences because of these works implies the inequality of cultural rights relations.

### 2.4. Online Movie Reviews

Films tell stories by audiovisual means. When the audience becomes immersed in them, they will empathize with the characters and project them onto themselves, comprehending and accepting the film from their point of view [42]. The audience then presents these feelings in a film review, which is an analysis and commentary on the film’s content, or, in other words, a study and interpretation of the form and content of the film [43]. These reviews provide good raw material for researchers. At the same time, audiences from different countries give different reviews because there are cultural differences in the emotional reactions evoked by films [44]. Therefore, the specific types of content users share are largely influenced by cultural values [45].

With the rapid development of the Internet, online film review websites have gained popularity. It reflects the rise of popular film criticism and the new characteristics of the film review system in the digital era [46]. Online reviews, as a kind of electronic word of mouth (eWOM), are very important for consumer decision-making for which culture is an important determinant [47]. These personal interactions also play a key role in product selection and dissemination [48]. Previous studies have compared online film reviews from different cultural backgrounds to explore whether they reflect the real or perceived quality of products [49]. There are also online movie review sites where one can arrange and compare film genres and audience gender [50], genre strengths, and Chinese and Western differences, and thus, contribute to cross-cultural communication [51].

## 3. Research Design and Results

### 3.1. Research Questions

Through the literature review, we found that Chinese culture and images of Chinese people in Western film and television media are becoming more diversified. However, previous studies mainly focused on the cross-cultural communication of local cultural products, while the research on the acceptance of cultural works with Chinese elements in foreign media in consumer countries is insufficient. At the same time, under the dominance of Western aesthetic values, Chinese audiences have always held a relatively negative and dissatisfied attitude towards such cultural hybridity. Judging from the Douban score alone, *Turning Red* seems to have achieved a breakthrough in this predicament. However, further research is needed to verify whether the Chinese audience’s attitude toward the hybrid works of Chinese and Western cultures has changed. Therefore, we propose the following research questions for this paper:

RQ1: What is the behavior of the Chinese audience’s receptive attitude towards *Turning Red*? What are the reasons behind it?

RQ2: Does this attitudinal behavior of the audience indicate a structural change in the hybridization of Chinese and Western cultures? What insight does this provide for the creation of cross-cultural products?

### 3.2. Methods

As grounded theory advocates, we will go into the problem without any presuppositions, generalize experience through sources, and finally rise to theory. Therefore, it is suitable for the exploration of new problems, new phenomena, and new fields. Glaser and Strauss pioneered grounded theory [52]. After continuous development, grounded theory is mainly derived from three schools. One is the classical grounded theory led by Glaser, which aims to explain a pattern of behavior. The second is the “programmed grounded theory”, proposed by Strauss and Juliet Corbin, based on positivism [53]. Then, the third theory is Kathy Charmaz’s constructivist grounded theory [54]. Most of the applied research practices of grounded theory use procedural grounded theory. Programmatic grounded theory is divided into three steps: open coding, axial coding, and selective coding.

### 3.3. Sample Collection: Online Film Review Website and Open-Ended Questionnaire

Douban.com is a UGC (user-generated content) website with great characteristics in China in the Web 2.0 era. It is also a book- and video-sharing platform, frequently used and widely recognized by domestic users. Viewers who review a movie on Douban.com can rate it between one and five stars, and eventually the platform will convert it into a rating out of ten through a specific algorithmic mechanism. In addition, viewers can also write short reviews or movie reviews to express their views and feelings about the movie. This time we used crawler software to collect short movie reviews of the movie *Turning Red* on Douban, and we finally obtained 220 short reviews and 661 movie reviews, totaling 631,622 words. Among these movie reviews, there were nine one-star movie reviews, 21 two-star movie reviews, 181 three-star movie reviews, 189 four-star movie reviews, and 261 five-star movie reviews.

Douban’s movie reviews are the main data source for this study, but to compensate for the lack of primary data and professional opinions, additional data from other sources collected to supplement this study. First, the questionnaire was released on the Internet, and second, the movie reviews from Mtime.com were collected. The questionnaire questions mainly involved basic information about the subjects, their rating of *Turning Red* (1 to 5), keyword descriptions, and an evaluative text. For the very few respondents who did not know about *Turning Red*, we invited them to describe their views on similar films. We finally obtained 77 valid records, totaling 16,266 words. Mtime.com is the world’s second largest film and TV database, second to IMDb. It aims to be “the most professional film and TV series and filmmaker database in China”. Compared with Douban, Mtime.com attracts more professional movie critics, so the movie reviews on Mtime.com are more professional. This study collected 151 movie reviews of *Turning Red* on Mime.com, including 16 long and 135 short movie reviews, totaling 16,424 words.

The collection of triangulated data provides a more comprehensive source while also cross-validating the reliability of the study. It should be noted, however, that the source of this study was derived from the prominence of the Douban ratings of *Turning Red* and the desire to verify through further research whether viewers’ scoring behaviors reflect whether they changed their views on such films, so Douban reviews remain the primary source for this study, while the other two data categories are used as secondary sources. Combining the data from the three parties, this study obtained a total of more than 664,000 words of textual material. Detailed information about the data has been shown in Table 2.

### 3.4. Coding

#### 3.4.1. Open Coding

We used Nvivo 12, a qualitative analysis tool, in the coding process. In the open coding stage, text materials should be constantly compared and summarized, and user comments should be abstracted into general concepts. In order to ensure the reliability and scientific validity of the coding, this study was conducted by two groups through communication and discussion. Then, the coding results were tested by the reliability index in Nvivo 12 to derive the corresponding kappa coefficients. Please see Appendix A for coding consistency results. Ultimately, this study summarized 162 basic concepts from the words and sentences of the textual material and then refined these concepts to arrive at 16 initial categories. The results of open coding are displayed in Table 3. 

#### 3.4.2. Axial Coding

In the axial coding stage, it is necessary to integrate the initial categories again, summarize the class and genus relations between them, and put the categories with similar characteristics under a certain logic together to form a new main category. Finally, the 16 initial categories can be classified into four main categories, namely “film production”, “theme type”, “cultural presentation” and “emotional tendency”.

#### 3.4.3. Selective Coding

During the selective coding phase, the categories were integrated. A “storyline” connects the key elements of how each category leads to the research question and their relationships. The key factor in our study is the audience’s attitude toward the film. By analyzing the existing core categories, we find that the emotional tendency is ultimately reflected in the audience’s attitude. What influences the formation of emotions is the presentation of film content, i.e., what we summarize as “film production”, “theme type”, and “cultural presentation”, which make up the cognitive layer of the audience in terms of sensibility, rationality, and intellectuality. Thus, we can induce a cognitive–emotional–attitudinal relational structure to form a selective coding. The results of the selective coding are displayed in Table 4.

#### 3.4.4. Theoretical Saturation Test

After the theoretical construction is completed, it is necessary to perform a theoretical saturation test to verify whether the current category and relationship structure adequately explain all the materials. In this study, about 1/10 of the original text was reserved for the theoretical saturation test, namely 60 film reviews and 20 short reviews from Douban, seven questionnaires, and 15 film reviews from Mtime. Through the analysis of these materials, it was verified whether new categories or new relationships could be formed outside the current main categories. The results showed that some new categories appeared, but the proportion was too low (less than three) to be statistically significant, so the theoretical model constructed in this study reached saturation.

## 4. Discussion

**Figure 1:**
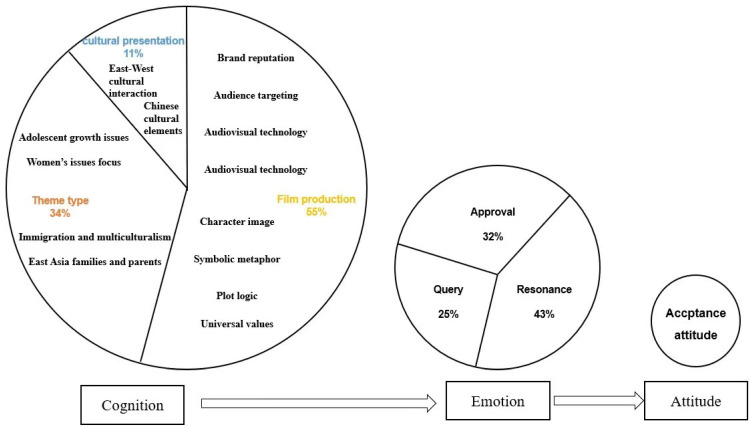
The mechanistic model of the audience-acceptance attitude formation process.

Based on the results of the cognitive–emotional–attitudinal relationship formed by the selective coding, we further clarified the mechanism model affecting the formation process of viewers’ receptive attitude behavior, as shown in Figure 1. It visually shows the proportion of viewers corresponding to the discussion part, and can also better explain how it affects the final acceptance attitude of viewers. We can discuss the core categories in this diagram in four dimensions.

### 4.1. Film Production: The Ability to Present a Film Story Is the Starting Point and Basis of Audience Evaluation

**Figure 2:**
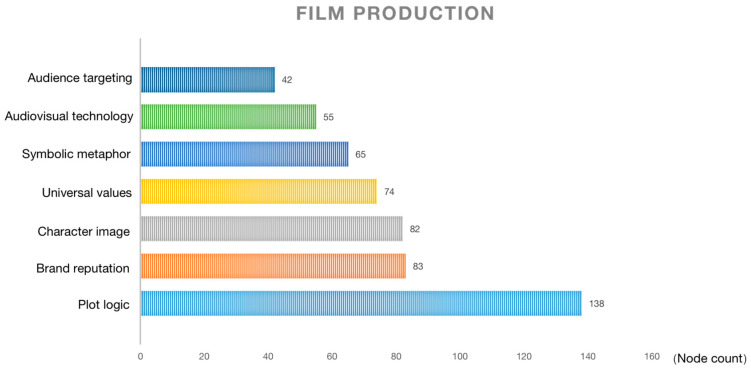
Film production.

The film production dimension is shown in Figure 2. As a comprehensive work of audio-visual art, the film tells the story through the screen, so the audience’s first concern is still how well the story is told. Film production comprises internal character image, symbolic metaphor, plot logic, universal value and external audience targeting, audiovisual technology, and brand reputation, which is the most discussed part of the audience.

In most Disney animation parenting styles, the father is more permissive, while the mother is more authoritative [55]. The film is also set up in such a way that the audience perceives the role of the father as flat and non-existent, and this role, which is outside the story, does not play any substantial role in advancing the film’s story in the early stages but becomes a key figure in resolving the conflict at the turning point of the story. This female dilemma, which still relies on men to unravel the setting, has triggered dissatisfaction among some viewers. However, the audience roles spark more discussion about the red panda; they think the image of the red panda is very cute. The red panda fully uses its visual advantage to please people and reaps the audience’s goodwill with its intuitive simplicity. Unlike the black and white colors of the giant pandas, the red panda gives a bright look on top of inheriting the cultural characteristics represented by China.

Animation allows for a freer use of metaphor, and in feature-length animation, a supporting metaphor is a creative metaphor that may itself be an image of the protagonist, such as the red panda in this film, whose features are linked to the story theme through a series of transformations [56]. Semantic complexity is one of the strengths of Pixar films [57], and many symbolic metaphors also appear in this film. The film’s title, “*Turning Red*”, suggests the change and development of the adolescent girl from physical to psychological dimensions, and it visualizes some indescribable emotions and desires.

As a family film, *Turning Red* is suitable for young and old alike, but some viewers believe that the film’s audience is relatively young due to the light and simple plot. Overall, audiences think that the film’s structure is complete and clear, but at the expense of story depth and intention. In addition, the audience discussion mainly focused on handling the ending. People felt that the resolution of the conflict was very abrupt, and the final ending of mother–daughter reconciliation and Meimei keeping the red panda inside her body was too idealistic and difficult to achieve in reality. This is also a common issue: the problems depicted in the film can only be solved in the film; the film cannot alter reality. These are typical Western-style universal values, advocating for the understanding of oneself and the pursuit of freedom. Such universal values can resonate widely across national and cultural boundaries.

Based on Disney’s and Pixar’s professional animation production team, the animation’s scene design, character design and music production are outstanding and have gained full recognition from the audience. In particular, the image rendering of pandas is such that one can almost feel the plush touch and is very realistic. This ensures the audience’s visual enjoyment while watching and helps them reach deep immersion. The brand reputation of Disney and Pixar attracted viewers to watch, reducing their decision time and ensuring the quality of the work. Audiences found the film to be the best combination of Disney and Chinese culture and Pixar’s first foray into the Chinese cultural world, which was invaluable.

In terms of the overall production of the film, the film is flawed, but it is a professional commercial film. The large audience discussion on this part also shows that the film’s storytelling ability is the starting point and basis for audience evaluation. The balanced performance in all aspects has laid the foundation for the word-of-mouth phenomenon around *Turning Red*.

### 4.2. Theme Type: Common Themes and Mainstream Issues Bridge Cross-Cultural Incommensurability

**Figure 3:**
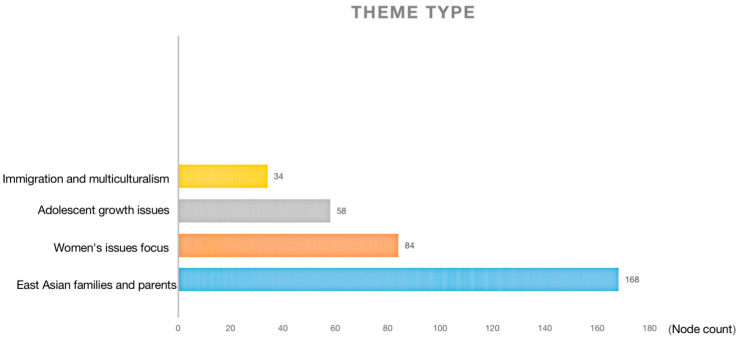
Theme type.

The theme-type dimension is shown in Figure 3. In cross-cultural communication, the incommensurability between different regions, countries and cultures leads to the difficulty of the culture with strong characteristics being popular worldwide. Content elements will aggravate the negative impact caused by cultural heterogeneity, while aesthetic elements can weaken such negative impact [58]. Therefore, the usual approach is to reduce the story’s cultural specificity while adding culturally specific aesthetic elements.

The story of *Turning Red* is about a 13-year-old Chinese Canadian girl named Mei, who undergoes subtle physical and psychological changes during her adolescent growth phase. As her gender identity and sense of individuality gradually come to the fore, she needs to re-address and adjust her relationship with her parents, friends, and society, and even re-learn herself. The director uses exaggeration to visualize this change by having the character transform into a giant red panda, and the film revolves around the retention or departure of this red panda inside Meimei. The story is not culturally specific; it concerns the growth issues that everyone experiences. As the protagonist has an immigrant family, it again indirectly shows this group’s portrayal, reflecting multiculturalism’s flourishing situation. Children of immigrants are frequently caught between two worlds, attempting to uphold their family’s honorable traditions while also ensuring that the opportunities of the new country, which their parents have worked so hard to arrive to, do not go to waste. Thus, the characters often project their parent’s fantasies, struggles, fears, and desires [59]. The film’s conflict focuses on Meimei and her mother, this exploration of the mother–daughter relationship touches on both women’s issues and parent–child relationships.

In recent years, Disney has actively improved gender representation in their animated films, and the power distance between male and female characters has been narrowed. Being a wife and mother is no longer the only way for women to bring honor to their families [60]. At the same time, the situation that men are the main characters and are depicted as more powerful [61] has also changed, which is the result of the widespread popularity of feminism around the world. The audience comes to believe that Meimei and her mother redeem each other, and Meimei and her female friends help each other, all of which reflect female power. The destructive force displayed by the red panda is a resistance to traditional characteristics that require women to be patient, sweet, understanding, and gentle.

The story of Meimei and her mother is also an in-depth reflection on East Asian family relationships, as one of the most popular short reviews said, “It feels like only East Asians can empathize because we all need to apologize for failing our mother’s expectations”. Viewers generally agreed that this is a very typical Eastern-style family parenting model. It involves parents who are overly expectant, overly caring, and overly controlling of their children. The parents’ very heavy love pressures the child to grow up according to the parent’s wishes, but the child loses themselves.

It can be seen that this story integrates common themes and mainstream issues, and the creator shows his acceptance of mainstream ideology with the help of and use of media platforms [62]. These problems are faced by all regardless of race or skin color. The solutions in different cultures do not show conflict, but try to provide us with references from different perspectives. This kind of content without cultural particularity bridges the incommensurability of cross-cultural communication.

### 4.3. Cultural Presentation: Audiences Lack Conscious Perception and Judgment Standards for Cultural Hybridity

**Figure 4:**
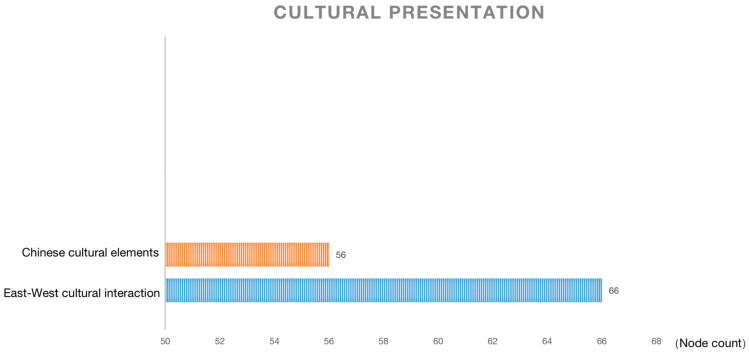
Cultural presentation.

The cultural presentation dimension is shown in Figure 4. Surprisingly, there is not much discussion of the presentation of culture in the film. Part of the discussion is about presenting Chinese cultural elements in the film, such as pandas, bamboo, courtyard houses, Chinese food, cheongsam, and others, or cultural connotations, such as the traditional Chinese filial piety culture and the Chinese family’s emphasis on unity. Since the director and scriptwriter of this film are of Chinese descent, the reflection of Chinese culture is true and objective, which reduces the risk of cultural misuse and is generally recognized by the audience.

The other part is evaluating the cultural interaction between East and West. The audience believes that Chinese and Western cultures are integrated, presenting a Hollywood-style Chinese story. Western cultural values dominate, while Chinese culture is only present in the background. Some viewers believe that it is a cultural conflict or even a cultural invasion, so they question whether the film’s content is biased, secretly promoting Western culture while devaluing Eastern culture. In fact, the application of Chinese culture in the film is not only in the background, and the interaction between Eastern and Western cultures is subtly shown at some important turning points in the plot.

For example, Meimei’s family is matriarchal, and every woman in the family turns into a red panda when she reaches a certain age. This ability was inspired by their ancestor Xinyi’s role in protecting the family during the ancient war period and has been passed down from generation to generation. It can be seen that the red panda here is a powerful female. However, the film does not attribute the source of this female power to the social ethos, namely the popularity of the feminist movement (although it originates from this). However, it describes it as a “mysterious power” using the narrative mode of ancient Chinese mythology.

Another example is the story ending, in which Meimei makes a different choice from the women in her family by keeping the red panda inside. The film acknowledges Meimei’s approach but does not criticize the elders for this “conservative” approach. The choice of keeping the red panda or discarding the red panda is personal; there is no better or worse, and everyone can still experience a happy ending while respecting differences.

The film does not advocate for the subordination of one culture over another, which is the interactive negotiation that cultural hybridity emphasizes. Cultural hybridity is related to two key fundamental conditions of globalization: de-territorialization and re-territorialization [63]. Therefore, local cultures are not reproduced intact but are somehow deformed in their interaction with global cultures. The immigrant groups that are the film’s focus are often “hybridity”, as they meander through different cultural worlds, enjoying the diversity of cultures that open their eyes and minds. Meanwhile, at the same time, they find the boundaries of their identities blurred and existing in a kind of “outside” space [64], which is what Homi Bhabha calls the “third space”. It is not the same as mingled culture, but a new cultural field, and thus, its similarity to the original culture cannot be measured. Moreover, cultural hybridity is not the average participation of two or more cultures, so it is not reasonable to evaluate the proportion of a certain culture but rather to analyze it in a specific historical context. From the audience’s descriptions, they do not possess a conscious awareness of cultural hybridity, nor do they have criteria for judging it.

### 4.4. Emotional Tendency: Audiences Pay More Attention to Individual Emotions Than Collective Cultural Performance

**Figure 5:**
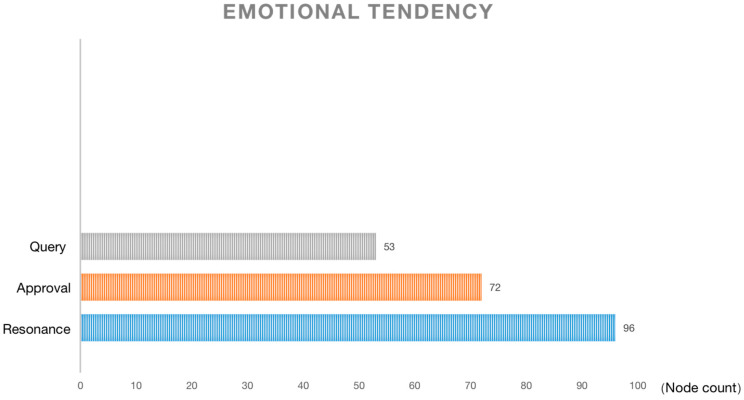
Emotional tendency.

The emotional tendency dimension is shown in Figure 5. It can be seen that the main emotion of the audience is resonance, followed by identification and, finally, questioning. The majority of the resonance is due to the audience’s self-projection. That is, when seeing the behavior and situation of the characters in the film, they will unconsciously project themselves into the situation as well [65]. Movies, like books, can enhance one’s mental capacity to understand others better [66]. Many comments from the audience indicated that they saw themselves in Meimei, so it was very easy to empathize with the characters in the film, and thus, resonate with them. As the child, she is the submissive one in the parent–child relationship. As a woman, she hides her true self under social rules. As a teenager, she needs friends and socialization to connect with society while growing up. Meanwhile, as a descendant of immigrants, she must struggle to find an identity in multiple cultures. This makes the audience able to identify with her and find similar life experiences. This cultural identity is more likely to appeal to a global audience than the emphasis on cultural hybridity would [67].

Part of the audience’s approval came from the affirmation of the film production, considering the film’s smooth plot and innovative setting. The other part is the approval of the cultural performance. The audience believes that the creative team has successfully managed both cultures, East and West, to integrate fully. The presentation of Chinese culture is no longer derogatory, but rather accurate. The audience also questioned the presentation of culture, saying that the East and West are unequal and that the film promotes Western educational methods while belittling and satirizing Eastern educational concepts. At the same time, the stereotypical portrayal is still present. Of course, from the viewers’ descriptions, the “stereotype” here is not exactly a pejorative term. It is more intended to express that the Chinese elements in the film are typical rather than new, such as the Chinese students always “wearing glasses”, “excelling in math”, and others.

Overall, the audience’s focus on resonance in this story is firstly about self-growth and secondly about the focus on Chinese culture and the interaction between East and West. The audience is more concerned with personal emotions than with collective cultural expressions. Culture is not the narrative’s core; the film focuses on specific people rather than generalized cultural symbols. The characters in the film are not used to promote a culture; rather, the more accurate and objective representation of culture appears to be the icing on the cake, and the story could be told in any skin color.

### 4.5. The Discussion of the Research Question

**Figure 6:**
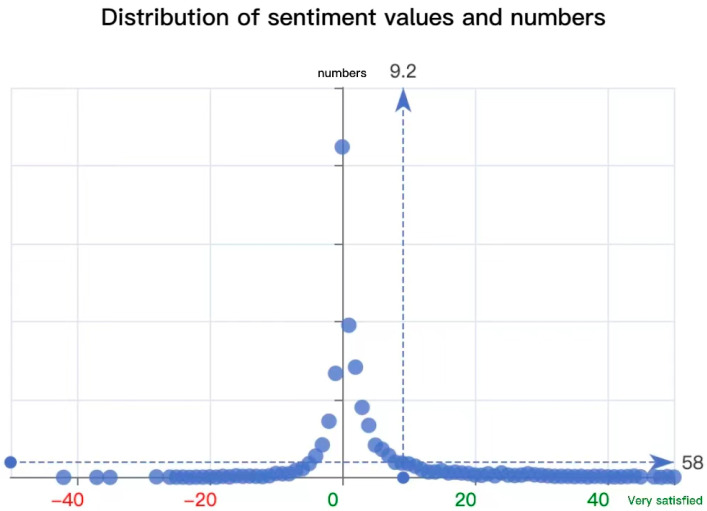
Sentiment distribution of the film reviews of *Turning Red*.

Based on the above discussion, we will try to answer the two questions raised at the beginning of the study. For RQ1, what is the behavior of the Chinese audience’s receptive attitude towards *Turning Red*? What are the reasons behind it? We discovered that cognitive-to-affective feedback is predominantly positive, and that the acceptance attitude is naturally oriented. We performed sentiment analysis on all the texts with the help of fenci.weiciyun.com, and a total of 4770 valid entries were filtered out. These sentiment words were assigned a value between −3 and 3, with values less than zero being negative and greater than zero being positive. The final relationship between the sentiment value and the number distribution was obtained, as shown in Figure 6. Among them, a positive sentiment was the most predominant, and the most concentrated sentiment value was 9.2 points in the positive direction. Thus, it is clear that the positive sentiment is much greater than the negative sentiment in the audiences’ evaluation of *Turning Red*. Correspondingly, it has indeed created a more positive attitude toward in China.

**Figure 7:**
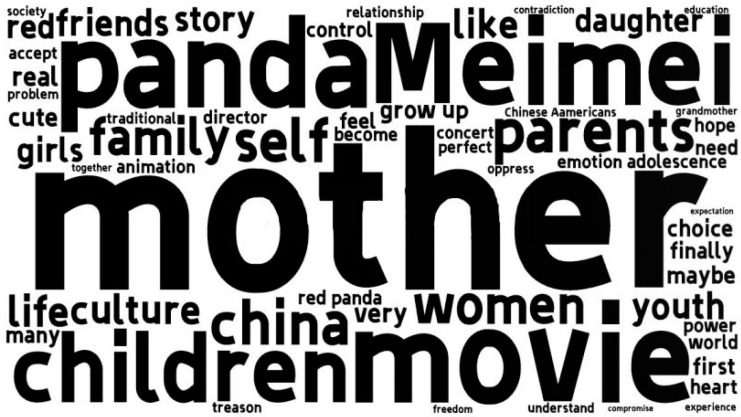
High-frequency word clouds in the film reviews and short reviews of *Turning Red* on Douban.

What is of concern is that this attitude of acceptance is not rooted in how skillfully the film balances East and West, or how East–West cultural hybridity achieves equality in the true sense of interaction. Rather, the film inspires individual, not collective, emotion in the audience. *Turning Red* achieves cultural identity, not a collective national cultural identity, but rather an identity about individual growth experiences. This we can verify with the help of the high-frequency word cloud in the original text. The results of high frequency words are shown in Figure 7. It can be seen that the audience’s discussion focused on the parent–child relationship issues directed by “mother”, “child”, “family”, and “parents”, followed by the attention to adolescents and women, and the culturally related issues came even later. Compared with cultural values, it is easier for personal real growth experience to obtain cross-cultural resonance. Cultural heterogeneity reflects the degree to which a film represents a particular culture [68]. Although this film has a regional cultural representation, it is not culturally heterogeneous, and thus, has attracted a wide audience.

As for RQ2, does this attitudinal behavior of the audience indicate a structural change in the hybridization of Chinese and Western cultures? What implications does this have for the creation of cross-cultural products? We do not think so. This inherent structure means that China provides surface cultural symbols, and the West provides core cultural values. Indeed, compared with previous works, *Turning Red* represents a certain progress. It respects cultural differences, and even though the final direction of the story is to conform to Western cultural values, it does not devalue Eastern values and even advocates for Chinese cultural values in some places. However, these expressions are so subtle that it is difficult for the audience to recognize them. Moreover, the extent of such changes is far from structural. Unlike cultural imperialism, cultural hybridity takes a relatively optimistic view of the flow of global cultures. Cultural hybridity only sometimes lies in the ideal non-power zone [69]. There is a constant struggle between authenticity and commodification [70]. The idealized two-way flow envisioned by cultural hybridity is difficult to achieve under inherent cultural capital inequality, or it is always difficult to achieve a balanced state of two-way flow.

### 4.6. Research Implications

This study actually showed that in global cultural exchange, national identity and cultural hybridity exist simultaneously [71]. However, there are differences between the two, and in cultural hybridity, the original national identity will make some adjustments to adapt to the new environment. Except for the group in this cultural space, other groups are unaware of this difference in time. This is why Chinese audiences have not been very positive about films with Chinese and Western cultural hybridity. This is because they assess whether the Chinese cultural elements expressed in these films are consistent with the local Chinese culture without paying attention to the fact that they are not completely equivalent to the original culture, which needs to be seen with a new evaluation scale and vision.

From a business and management perspective, this has implications for both audience consumption behavior and film and television creative guidance. First of all, in terms of audience consumption behavior, on the one hand, we noticed that the audience did not realize the difference between the hybrid culture and the original culture, so we should pay attention to guide and distinguish it. That is to say that we should set up different cognitive standards to treat culture, so as not to fall into the limitations of cultural cognition, but to see the fluidity and plasticity of culture. On the other hand, the audience’s focus on personal emotion shows that they attach importance to their own emotional value in consumption behavior. That is to say that in a real consumption situation, the audience is more likely to be moved by the resonance and empathy generated by personal emotions.

Based on this, we think that several points can be used for reference when applied to specific cross-cultural creations.

First of all, the creation of the story itself takes precedence over everything else. Disney and Pixar have established teams of writers and directors, and their technical skills are exceptional. Such a strong guarantee allows the production team to accurately convey the story idea to the audience and give them a wonderful visual experience. Therefore, whether it is a hybrid of collective culture or an expression of individual emotions, high quality creation is the most important prerequisite.

Secondly, for cross-cultural works, creators should abandon the blind pursuit of cultural symbols and choose to focus on specific “people”, specific problems, and a specific cultural content. To gain international cultural confidence, cultural export should not blindly display the excellent traditional culture in the local culture. For the audience, it seems too idealistic and impractical. The narrative of grand cultural values should be implemented on a small and real level, and the existing problems should be sincerely discussed to trigger transnational resonance honestly.

Thirdly, *Turning Red* can objectively restore Chinese culture thanks to the cultural background of the directorial team, which has real experience growing up in Chinese families. This real experience in the cultural hybridity space improves the accuracy of cultural expression. The acquisition of cultural symbols is rapid and easy to perceive, but the culture’s deep connotations require the local culture’s subtle influence over the years. Creators need to be rooted in the real cultural soil, and only through long-term investigation and profound experience can they present the original local culture, which should not only be based on assumptions, let alone preconceptions and prejudices.

## 5. Conclusions

Based on a grounded study of the audiences’ reviews of Disney’s Pixar-animated film *Turning Red*, the audience behavior was to give positive reviews because it inspired resonant emotions in them, rather than changing Chinese audiences’ opinions on the level of Chinese cultural expression. Certainly, the producer’s experience in commercial production and their artistic ability, combined with the film’s focus on topical issues and common global themes, laid the foundation for the film’s critical acclaim. However, the status quo of Chinese and Western cultural hybridity has not achieved a major breakthrough and change. On the one hand, this implies that individual emotional tendencies are more likely to influence individual behavioral choices than the collective cultural atmosphere. On the other hand, it also shows that people in their own country are not sensitive to the cultural forms that result from blending their own culture with that of other countries. They still measure and evaluate according to their original cultural judgment standards.

### 5.1. Academic and Managerial Implications

The study also reaffirms the internal complexity and fluidity of the cultural hybridity theory itself, which makes it show a different dynamic from the original culture and makes the audience of the original culture seem confused in the process of consumption. The lack of audience awareness of cultural hybridity is an important reason why similar cultural products have not been well received in the past, but *Turning Red* makes up for this deficiency with personal life experiences that can cover almost all levels.

Correspondingly, we make suggestions for cross-cultural creation, arguing that we should start from the audience’s emotions, search for the demands that exist in individuals in the globalization situation, and strengthen the in-depth understanding and experience of cultural connotations. At the same time, we also call for establishing an evaluation scale for cultural hybridity that is different from that of local culture.

The understanding of Oriental culture among Chinese families mostly stagnates in the first generation of immigrants, who retain their cultural memories of their home countries and pass them on to their descendants in foreign lands. However, at the same time, Eastern culture itself is still constantly changing and developing. Hence, the Eastern culture they exhibit is incompatible with the real Eastern culture today. The parent–child relationship, on the other hand, is exceptionally consistent, making the subject matter resonate even when time and space are dislocated. The parent–child relationship in the movie has an idealized ending in a family-friendly atmosphere, whereas in real life, parental social support is an important factor influencing intergenerational cultural conflicts [72] and maintaining one’s own traditional culture; that is, identification with filial piety is an important safeguard to prevent all conflicts [73]. The specific manifestation of intergenerational conflict may vary from one era to another, but it always exists and will persist. This cultural memory, which resembles the collective unconscious, allows the audience to be influenced by the collective psychological experience in the reception process.

### 5.2. Research Reflection and Prospect

No platform’s data can avoid limitations. For example, most of the Douban users are young people, between 20 and 30 years old, with a higher education background. It cannot fully reflect and represent the views and feelings of all of the movie’s viewers. Based on this situation, we refined and enriched the research data from two more sources: movie reviews from Time.com and an open-ended questionnaire collection. Nonetheless, the study may still leave out the opinions of a portion of the audience that is not used to using social networks, which is both the potential and limitation of this study.

This study adopts a grounded theory approach, which places emphasis on original material and on the bottom-up step-by-step generation of a theoretical framework. As a result, it is not suitable for determining theoretical frameworks in advance, but rather for dealing with new and unknown problems As in the case of the film *Turning Red* in this study, which has achieved a different evaluation from previous culturally hybrid films here in China, in the face of this new phenomenon and the possible reasons behind it, it is necessary to adopt a grounded theory to extract concepts and structures from the audience’s evaluation. We have combined the use of the Nvivo software with grounded theory to make it easier to operate and to facilitate a series of visualizations directly from the software technology, making the results of the study more visible. The combination of grounded theory and the Nvivo software is the contribution of this study in terms of theory and methodology.

Finally, the novelty of this study can be seen in two points. One is that grounded theory itself is widely used in anthropological or sociological research, but this study introduces it into the research of cross-cultural communication, which is an innovation at the methodological level. The second is to explore the profundity and complexity of the original theories of cultural hybridity, and whether the audience’s cognitive standards have changed simultaneously when their own culture has become “other” after being hybridized. We hope obtained different results from the novel phenomenon of *Turning Red* and study it to see if it changes the original theory, which is an innovation at the theoretical level.

This study has two shortcomings. First, our study is on Chinese and Western cultural hybridity. However, cultures are extremely different, and whether this can be realized in other forms of cultural hybridity needs further verification. Second, Douban’s short reviews are selected and randomly displayed based on time and popularity, so the data we obtained are limited due to the platform’s limitations. Although we also investigated the full movie reviews, which are longer and more informative, incomplete access to short reviews still has certain shortcomings.

Future research prospects can be derived from both horizontal and vertical aspects. On the one hand, we can focus on Chinese audiences’ acceptance behaviors of other Chinese and Western cultural hybridity products, such as fantasy novels, which are also products of cultural hybridity. However, Chinese audiences seem to love them very much. There are also other art genres, such as music and drama, or games, known as the “Ninth Art”. On the other hand, it is possible to compare the different reactions and behaviors of Western and Chinese audiences to the same work to study how cultural hybridity affects the local culture, particularly in the cultural regions involved.

## Figures and Tables

**Table 1:** Comparison between the scores of Douban and IMDb for Chinese and Western cultural hybridity films in the past five years (Statistics as of June 2022).

The Name of the Film	Release Date	Douban Score (Number of Reviewers)	IMDb Score (Number of Reviewers)	The Gap
*Crazy Rich Asians*	2018	6.0 (Reviewed by 112,000 people)	6.9 (Reviewed by 165,000 people)	−0.9
*The Farewell*	2019	7.2 (Reviewed by 87,000 people)	7.5 (Reviewed by 61,000 comments)	−0.3
*Mulan*	2020	4.8 (Reviewed by 302,000 people)	5.7 (Reviewed by 145,000 people)	−0.9
*Shang-Chi and the Legend of the Ten Rings*	2021	6.1 (Reviewed by 186,000 people)	7.4 (Reviewed by 346,000 people)	−1.3
*Turning Red*	2022	8.2 (Reviewed by 205,000 people)	7.0 (Reviewed by 83,000 people)	+1.2
*Everything Everywhere All at Once*	2022	7.7 (Reviewed by 332,000 people)	8.3 (Reviewed by 128,000 people)	−0.6

**Table 2:** The statistical breakdown of study data sources.

Data Source	Category	Amount	Word Count	Serial Number
Douban.com	One-star movie reviews	9 articles	5916	Sample one
	Two-star movie reviews	21 articles	19,055	Sample two
	Three-star movie reviews	181 articles	144,246	Sample three
	Four-star movie reviews	189 articles	194,322	Sample four
	Five-star movie reviews	261 articles	242,538	Sample five
	Short reviews	220 items	25,545	Sample six
Mtime.com	Movie reviews	151 items	16,424	Sample seven
Questionnaire	Open-ended questions	77 copies	16,266	Sample eight

**Table 3:** Open coding.

Number	The Initial Category	Example of Original Material
1	Resonance	“Actually, seeing myself in the movie.”
2	Brand reputation	Pixar’s animated movies never let people down
3	Audiovisual technology	“The picture quality and music quality are very high, the production is quite well.”
4	Audience targeting	“A movie for adults and children”
5	Character image	“All male characters are flattened in the treatment.”
6	Symbolic metaphor	“Red = menarche for women, Red moon, Red hairy panda, Chinese Red”
7	Plot logic	“The ending feels a bit far-fetched and overly idealized.”
8	Universal values	“Get rid of others’ expectations and find your true self.”
9	Adolescent growth issues	“Something inherent in the process of growing up and in the search for self”
10	Women’s issues focus	“Focuses entirely on the female psyche, female growth and the journey of repression by discipline.”
11	Immigration and multiculturalism	“Second-generation immigrants, especially Asians, are gradually becoming a creative motif.”
12	East Asian families and parents	“Very typical East-style family parent–child template presentation”
13	Chinese Cultural elements	“Chinese cultural elements throughout the story”
14	East–West Cultural interaction	“Oriental Confucian films, but with a distinctly Western individualism.”
15	Approval	“A perfect collision of Chinese culture and Hollywood movies”
16	Query	“The image of the extreme mother appears stereotypical when she is of Chinese descent.”

**Table 4:** Selective coding.

Relational Structure	Connotation
Cognition–Emotion–Attitude	The audience’s perception of the film conveys emotional tendencies, which determine their acceptance of the film.
Film production: Cognition–Emotion	The sensual, rational perceptions of storytelling and creative quality are presented inside and outside the film.
Theme type: Cognition–Emotion	How the audience perceives the presentation of Chinese culture and the interaction between Eastern and Western cultures in the film belongs to the perception of intellectuality.
Cultural presentation: Cognition–Emotion	How does the audience view the presentation of the interaction between Chinese culture and Eastern and Western cultures in the film as intellectual cognition.
Emotional tendency: Emotion–Attitude	The audience’s feelings are generally presented as approval, resonance, query, leading to the final attitude of acceptance or resistance.

## Data Availability

Publicly available datasets were analyzed in this study. These data can be found here: https://movie.douban.com/subject/35284253/ (accessed on 28 November 2022) and http://movie.mtime.com/269513/ (accessed on 28 November 2022).

## Appendix A. The Results of Coding Consistency Test

To ensure the reliability of the coding, other members of the research team were invited to code independently after detailed training and adequate communication. At the end of coding, reliability percentage tests were conducted at NVivo 12 through coding comparisons.

**Table A1:** Results of coding consistency tests.

Main Categories	Source of Material	Kappa	Consistency (%)	Inconsistency (%)
Film production	Douban.com	0.57	96.38	3.63
	Mtime.com	0.63	97.26	2.74
	Questionnaire	0.65	97.86	2.14
Theme type	Douban.com	0.71	96.29	3.71
	Mtime.com	0.62	94.58	5.42
	Questionnaire	0.75	96.52	3.48
Cultural presentation	Douban.com	0.78	98.36	1.64
	Mtime.com	0.81	97.84	2.16
	Questionnaire	0.77	96.19	3.81
Emotional tendency	Douban.com	0.56	93.86	6.14
	Mtime.com	0.82	95.17	4.83
	Questionnaire	0.74	95.39	4.61

The Kappa coefficient is a measure used to measure consistency and is calculated as follows:

$$Κ=\frac{P_{o}-P_{e}}{1-P_{e}}$$

The results of the kappa measurements showed that the kappa values had five levels of agreement: mild agreement (0.0–0.20), general agreement (0.21–0.40), moderate agreement (0.41–0.60), substantial agreement (0.61–0.80), and almost complete agreement (0.81–1).
